# Inflammation-induced inhibition of chaperone-mediated autophagy maintains the immunosuppressive function of murine mesenchymal stromal cells

**DOI:** 10.1038/s41423-019-0345-7

**Published:** 2020-01-03

**Authors:** Jie Zhang, Jiefang Huang, Yuting Gu, Mingxing Xue, Fengtao Qian, Bei Wang, Wanlin Yang, Hongshuang Yu, Qiwei Wang, Xin Guo, Xinyuan Ding, Jina Wang, Min Jin, Yanyun Zhang

**Affiliations:** 1 CAS Key Laboratory of Tissue Microenvironment and Tumor, Shanghai Institute of Nutrition and Health, Shanghai Institutes for Biological Sciences, Chinese Academy of Sciences, Shanghai, China; 2 Pediatric Institute of Soochow University, Institutes for Translational Medicine, Soochow University, Suzhou, China; 3 Shanghai Institute of Immunology, Shanghai Jiao Tong University School of Medicine, Shanghai, China; 4 Department of Urology and Shanghai Key Laboratory of Organ Transplantation, Zhongshan Hospital, Fudan University, Shanghai, China

**Keywords:** chaperone-mediated autophagy, mesenchymal stromal cells, immunosuppressive capacity, inflammatory microenvironment, Immunosuppression, Chaperone-mediated autophagy

## Abstract

Macroautophagy has been implicated in modulating the therapeutic function of mesenchymal stromal cells (MSCs). However, the biological function of chaperone-mediated autophagy (CMA) in MSCs remains elusive. Here, we found that CMA was inhibited in MSCs in response to the proinflammatory cytokines interferon-γ (IFN-γ) and tumor necrosis factor-α (TNF-α). In addition, suppression of CMA by knocking down the CMA-related lysosomal receptor lysosomal-associated membrane protein 2 (LAMP-2A) in MSCs significantly enhanced the immunosuppressive effect of MSCs on T cell proliferation, and as expected, LAMP-2A overexpression in MSCs exerted the opposite effect on T cell proliferation. This effect of CMA on the immunosuppressive function of MSCs was attributed to its negative regulation of the expression of chemokine C-X-C motif ligand 10 (CXCL10), which recruits inflammatory cells, especially T cells, to MSCs, and inducible nitric oxide synthase (iNOS), which leads to the subsequent inhibition of T cell proliferation via nitric oxide (NO). Mechanistically, CMA inhibition dramatically promoted IFN-γ plus TNF-α-induced activation of NF-κB and STAT1, leading to the enhanced expression of CXCL10 and iNOS in MSCs. Furthermore, we found that IFN-γ plus TNF-α-induced AKT activation contributed to CMA inhibition in MSCs. More interestingly, CMA-deficient MSCs exhibited improved therapeutic efficacy in inflammatory liver injury. Taken together, our findings established CMA inhibition as a critical contributor to the immunosuppressive function of MSCs induced by inflammatory cytokines and highlighted a previously unknown function of CMA.

## Introduction

Autophagy takes its name from the Ancient Greek “autóphagos”, meaning “self-eating”. As its name implies, autophagy degrades intracellular components, such as proteins and organelles inside lysosomes and, as a result, contributes to cellular homeostasis, quality control, and maintenance of energetic balance.^1^ Three main types of autophagy have been described, macroautophagy, microautophagy, and CMA, which differ based on the mechanisms that mediate the delivery of cytosolic cargo to lysosomes for degradation.^2^ CMA was the first studied autophagy form which has selectivity for its target proteins. To be CMA substrates, proteins must contain a “KFERQ”-like motif in their amino acid sequence, and this motif binds to a cytosolic chaperone heat shock 70 kDa protein 8, which brings the substrate protein to the lysosomal surface. The CMA-related lysosomal receptor LAMP-2A forms a multimeric protein complex channel for translocation of the substrates into lysosomes. LAMP-2A is the rate-limiting component during this process and thus is often used as a specific target to intervene in the activity of CMA.^3^

As a result of its selectivity for substrates, CMA participates in multiple cellular functions via degradation of particular proteins, in addition to the common features that all autophagy types share. For example, CMA is necessary for CD4^+^ T cell activation because it selectively degrades two negative regulators of T cell receptor signaling, the E3 ubiquitin-protein ligase Itchy homolog and regulator of calcineurin 1.^4^ Although many studies have investigated the function and regulation of CMA, compared with what is known about macroautophagy, there is still much to learn about CMA.

MSCs have attracted much attention for their ability to regulate inflammatory processes.^5^ The mechanisms by which MSCs exert their immunomodulation are multifaceted. In an inflammatory environment, MSCs apply indirect regulatory effects to T cells and potentially B cells via regulating the response of innate immune cells, thus alleviating inflammation. Our previous work demonstrated that in inflammation-mediated spontaneous abortion models, treatment with MSCs inhibited the proliferation of CD4^+^ T cells by inducing the decidual macrophage switch to the M2 phenotype, which led to the abortion relief.^6^ In addition, MSCs also exert direct effects on T cells through the production of immunosuppressive molecules, such as NO, indoleamine 2,3-dioxygenase, prostaglandin E2, transforming growth factor β (TGFβ), heme oxygenase 1, and leukemia inhibitory factor. This effect occurs only when T cells are in close proximity to MSCs, and so chemokines, such as CXCL9, CXCL10, and CXCL11 released by MSCs play a necessary role in recruiting T cells to the surroundings of MSCs.^7^ Studies of the interplay between inflammatory factors and MSCs have revealed that inflammation is crucial in empowering the immunomodulatory activity of MSCs.^8^ However, the mechanisms by which the inflammatory microenvironment controls the immunoregulatory function of MSCs are not well understood.

Our previous work demonstrated that in the inflammatory microenvironment, macroautophagy was activated in MSCs. The increased level of macroautophagy inhibits the immunosuppression of MSCs on T cells via decreasing prostaglandin-endoperoxide synthase 2 expression.^9^ Published studies demonstrated that when macroautophagy is abolished in the absence of autophagy related 5, CMA is significantly enhanced,^10^ and these results also suggest a balance between CMA and macroautophagy.^11^ Thus, we hypothesized that CMA also plays a role in regulating the function of MSCs.

Here, we found that CMA activation was inhibited in MSCs treated with the proinflammatory factors IFN-γ and TNF-α, and activated AKT induced by these factors contributed to this inhibition of CMA. When CMA was blocked, the immunosuppressive capacity of MSCs on T cell proliferation was significantly enhanced, characterized by increased expression of CXCL10 and iNOS. Hence, CMA-deficient MSCs presented better therapeutic efficacy in inflammatory liver injury. Our findings established CMA as a critical negative regulator of inflammation-induced immunosuppression by MSCs.

## Results

### The inflammatory factors TNF-α and IFN-γ induce CMA inhibition in MSCs

**Fig. 1:**
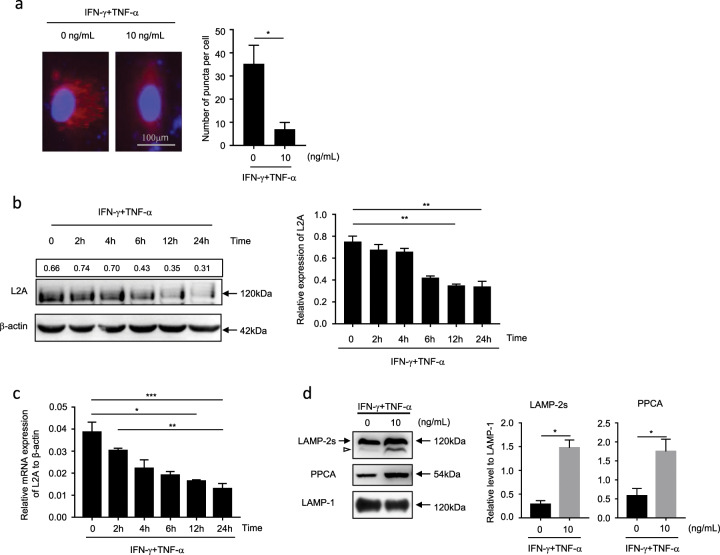
The inflammatory factors TNF-α plus IFN-γ induce CMA inhibition in MSCs. **a** MSCs stably expressing KFERQ-PA-mCherry-C1 were photoactivated by a 405-nm light and then treated with TNF-α plus IFN-γ (10 ng/mL each) for 24 h. The images were collected by a Zeiss fluorescence microscope. The number of red fluorescent puncta per cell was quantified. Scale bar: 100 μm. **b**, **c** MSCs were treated with TNF-α plus IFN-γ (10 ng/mL each), and mRNA and protein were collected at 0 h, 2 h, 4 h, 6 h, 12 h, and 24 h after treatment. Expression of LAMP-2A (L2A) was detected at the protein and mRNA levels using immunoblotting and quantitative real-time PCR, respectively. **d** Lysosomes from MSCs treated with/without TNF-α plus IFN-γ (10 ng/mL) for 24 h were isolated, and proteins were extracted for immunoblot analysis of all forms of LAMP-2 (upper) and PPCA (lower). The arrowhead indicates a previously identified truncated form of LAMP-2 lacking the cytosolic/transmembrane region. LAMP-1 was used as a control for normalization. The relative levels of the truncated forms of LAMP-2 and PPCA were quantified. The results are representative of three to six independent experiments and are presented as the mean ± s.e.m. Significant differences were analyzed by Mann–Whitney *U* test **a**, **d** or one-way ANOVA **b**, **c**, and are expressed as: *P < 0.05, **P < 0.01, and ***P < 0.001.

Proinflammatory factors, such as IFN-γ, TNF-α, and interleukin-1β, are necessary for inducing the immunosuppressive capacity of MSCs.^12^ To explore the role of CMA in the inflammation-induced immunosuppressive capacity of MSCs, we examined the CMA activation state in MSCs treated with/without IFN-γ plus TNF-α using the CMA reporter KFERQ-PA-mCherry1,^13^ which allowed for visualization of lysosomes as red fluorescent puncta when CMA was activated. The results showed that the number of red fluorescent puncta in IFN-γ plus TNF-α-treated MSCs was significantly decreased compared with that of the control cells (Fig. 1a). This indicated that proinflammatory factors induce CMA inhibition in MSCs. Then, we further detected the expression of LAMP-2A, a known marker of CMA, in MSCs upon IFN-γ plus TNF-α stimulation. Both the protein and mRNA levels of LAMP-2A decreased significantly in MSCs after stimulation with IFN-γ plus TNF-α (Fig. 1b, c).

In addition to de novo synthesis of LAMP-2A, which influenced CMA activity, changes in LAMP-2A degradation also play an important role in regulating CMA flux.^14^ To test the possibility that stimulation with proinflammatory factors accelerates the degradation of LAMP-2A, we isolated the lysosomes of MSCs treated with/without IFN-γ plus TNF-α for 24 h to detect the amount of protective protein/cathepsin A (PPCA), which is one of the main proteases that initiates the degradation of LAMP-2A and is inversely correlated with CMA activity.^15^ Additionally, the accumulation of a truncated form of LAMP-2A lacking the cytosolic and transmembrane regions was detected with an antibody against the luminal region of LAMP2, which indicated the degradation of LAMP-2A in lysosomal membranes.^14^ The results revealed that with IFN-γ and TNF-α treatment, the amount of PPCA in lysosomes, as well as the truncated form of LAMP-2A, was markedly increased (Fig. 1d), indicating that proinflammatory factors accelerated the degradation rate of LAMP-2A.

These data collectively suggest that CMA is downregulated in MSCs under inflammatory conditions due to the joint action of decreased de novo synthesis and enhanced degradation.

### CMA negatively regulates the immunosuppressive capacity of MSCs

**Fig. 2:**
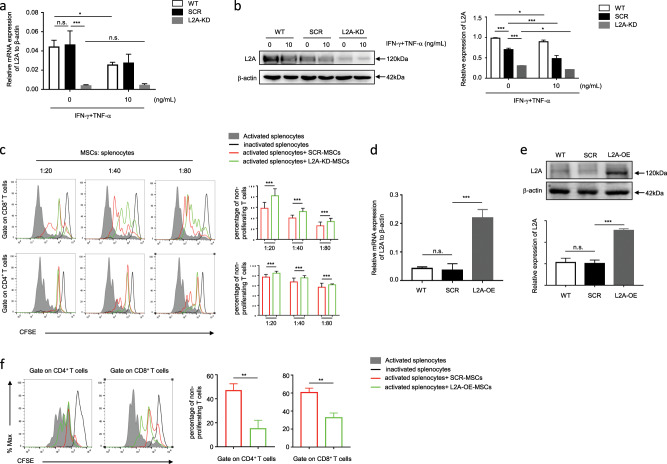
CMA negatively influences the immunosuppressive capacity of MSCs on T cell proliferation. **a**, **b** Wild-type MSCs (WT-MSCs) and MSCs transfected with lentivirus expressing shRNA targeting LAMP-2A (L2A-KD-MSCs) or scramble shRNA (SCR-MSCs) were treated with or without TNF-α plus IFN-γ (10 ng/mL each) for 24 h. mRNA and protein were collected, and LAMP-2A expression was measured by quantitative real-time PCR and immunoblotting analysis. **c** L2A-KD-MSCs and SCR-MSCs were pretreated with mitomycin C (50 ng/mL) for 4 h and then cocultured with CFSE-labeled splenocytes for 3 days in the presence of anti-CD3/CD28 antibodies at the indicated ratios. CD8^+^ and CD4^+^ T cells were stained for proliferation analysis by flow cytometry at the end of coculture, and the percentage of nonproliferating T cells is shown. **d**, **e** MSCs were infected with pLVX-IRES-zsGreen1-LAMP-2A (L2A-OE) or pLVX-IRES-zsGreen1 (SCR) lentivirus, and the GFP^+^ MSCs were sorted by FACS. Then, proteins and mRNAs of WT, SCR, and L2A-OE-MSCs were collected. The expression of LAMP-2A at the mRNA and protein levels was tested by quantitative real-time PCR and immunoblotting, respectively. **f** L2A-OE-MSCs and SCR-MSCs were pretreated with mitomycin C (ng/mL) for 4 h and cocultured with splenocytes labeled with CFSE, and activated by anti-CD3/CD28 antibodies for 3 days at a ratio of 1:20. CD8^+^ and CD4^+^ T cells were stained for proliferation analysis by flow cytometry at the end of coculture, and the percentage of nonproliferating T cells is shown. The results are representative of three to six independent experiments and are presented as the mean ± s.e.m. Significant differences were analyzed by Mann–Whitney *U* test **c**, **f**, one-way ANOVA **d**, **e**, or two-way ANOVA **a**, **b**, and are expressed as: *P < 0.05, **P < 0.01, ***P < 0.001, and n.s., no significance.

To further study the role of CMA in regulating the immunosuppressive capacity of MSCs, we knocked down LAMP-2A (Supplementary Fig. 1a) or overexpressed LAMP-2A in MSCs to examine their effects on T cell proliferation. As shown in Fig. 2a–c, LAMP-2A knockdown MSCs (L2A-KD-MSCs) suppressed the proliferation of activated T cells more efficiently than those infected with a lentivirus expressing scrambled short hairpin RNA (shRNA; SCR-MSCs). In contrast, LAMP-2A-overexpressing MSCs (L2A-OE-MSCs) had dramatically inhibited immunosuppressive effects on T cell proliferation (Fig. 2d–f). In addition, the experiments with MSCs cocultured with T hybridoma A1.1 cells further supported the negative role of CMA in the effects of MSCs on T cell proliferation (Supplementary Fig. 1b). These observations collectively identify a critical role of CMA in negatively regulating the immunosuppressive capacity of MSCs.

Proinflammatory factors not only enable the immunosuppressive function of MSCs but also inhibit the proliferation and induce apoptosis of MSCs, hence limiting their immunosuppression.^16,17^ Therefore, it is possible that CMA-mediated inhibition of MSC immunosuppressive capacity is due to CMA promoting cell death or repressing the proliferation of MSCs. However, there was no obvious difference in the proliferation between L2A-KD-MSCs and SCR-MSCs (Supplementary Fig. 1c). In addition, clone formation ability, which represents stemness and the proliferative capacity of MSCs, was not affected by LAMP-2A knockdown (Supplementary Fig. 1d). These results suggest that CMA negatively regulates MSC immunosuppression in a manner that is independent of cell death and proliferation.

### CMA inhibition promotes T cell recruitment to MSCs

**Fig. 3:**
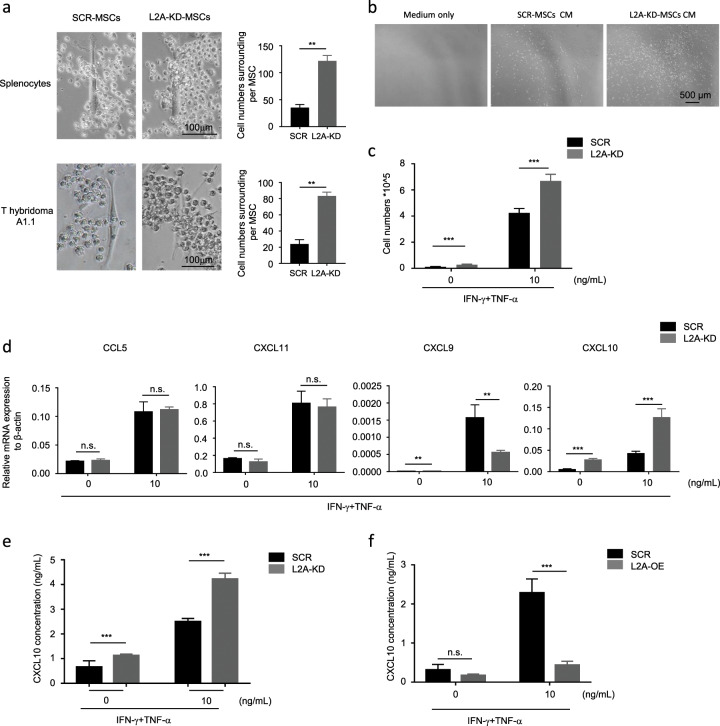
L2A-KD increases MSC recruitment of T cells by enhancing CXCL10 expression. **a** SCR-MSCs and L2A-KD-MSCs were cocultured with splenocytes or T hybridoma A1.1 cells in the presence of anti-CD3/CD28 antibodies at a ratio of 1:10 for 24 h. The extent of cell aggregation was observed and imaged microscopically. The number of splenocytes or T hybridoma A1.1 cells surrounding each MSC was quantified. **b** The culture medium (CM) of SCR-MSCs and L2A-KD-MSCs treated with TNF-α plus IFN-γ (10 ng/mL, respectively) for 24 h was collected. Then, this culture media were placed into the basolateral chambers of the transwell inserts (5 μm), with unconditioned medium used as the negative control, and T hybridoma A1.1 cells were placed in the apical chambers of the transwell system. Four hours later, the transwell inserts were removed, and the A1.1 cells that migrated into the basolateral chamber were observed and photographed. **c** The A1.1 cells that migrated into the basolateral chamber were counted, and the statistical analysis is shown. **d** SCR-MSCs and L2A-KD-MSCs were treated with or without TNF-α plus IFN-γ (10 ng/mL each) for 24 h. mRNA was collected, and the expression of CCL5, CXCL11, CXCL9, and CXCL10 was measured by quantitative real-time PCR. **e**, **f** SCR-MSCs, L2A-KD-MSCs, and L2A-OE-MSCs were treated with or without TNF-α plus IFN-γ (10 ng/mL each) for 24 h, and then the supernatant was collected to detect the concentration of CXCL10 using an enzyme-linked immunosorbent assay. Scale bar: 100 μm. The results are representative of three to six independent experiments and are presented as the mean ± s.e.m. Significant differences were analyzed by the Mann–Whitney *U* test **a** or two-way ANOVA **b**–**f**, and are expressed as **P < 0.01, ***P < 0.001, and n.s., no significance.

In the coculture system of MSCs with activated splenocytes or A1.1 cells, we found a greater number of recruited immune cells around L2A-KD-MSCs compared with that of SCR-MSCs (Fig. 3a). Time-lapse microvideography revealed increased migration of splenocytes toward L2A-KD-MSCs within 12 h of coculture initiation (Supplementary Movie 1). We hypothesized that CMA modulated the MSC recruitment of T cells. To this end, we then performed a transwell assay to examine the ability of L2A-KD-MSCs to attract T cells. As expected, L2A-KD-MSCs attracted more T cells into the target chamber (data not shown). The culture medium of L2A-KD-MSCs exerted the same effects (Fig. 3b, c), indicating that some MSC secretions were affected by CMA. It has been well characterized that chemokines, such as CXCL9, CXCL10, CXCL11 and CCL5, are necessary for MSCs to recruit T cells to their surroundings and then directly inhibit the proliferation of T cells.^7^ We then stimulated SCR-MSCs and L2A-KD-MSCs with IFN-γ plus TNF-α to detect the expression of these chemokines. The results revealed that LAMP-2A knockdown did not affect the gene expression of CXCL11 and CCL5, and even inhibited CXCL9 expression (Fig. 3d), whereas the expression of CXCL10 was notably enhanced at both the mRNA and protein levels (Fig. 3d, e). In contrast, L2A-OE-MSCs exerted the opposite effect on CXCL10 expression (Fig. 3f). Therefore, these data collectively suggest that CMA suppresses the expression of CXCL10 in MSCs, resulting in the reduced recruitment of T cells.

### CMA negatively regulates iNOS expression in MSCs

**Fig. 4:**
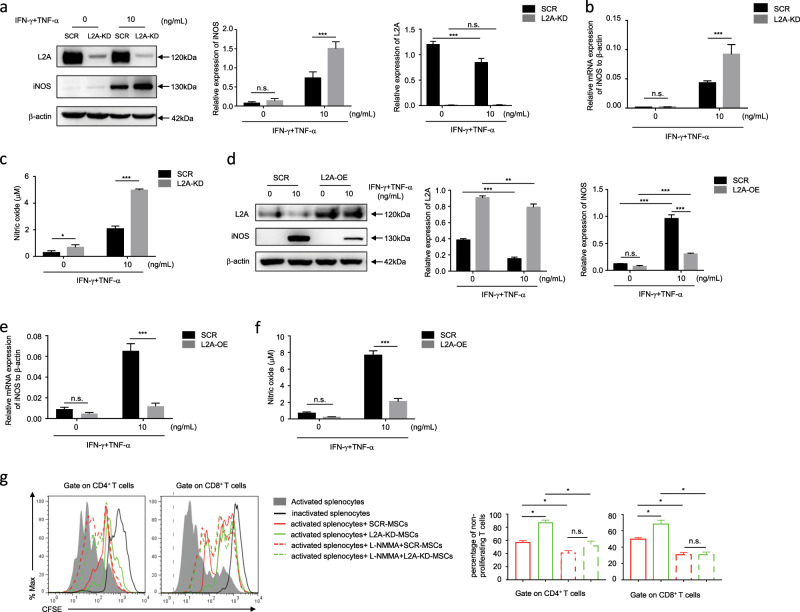
CMA inhibition significantly promotes iNOS expression in MSCs. **a**–**f** SCR-MSC, L2A-KD-MSCs, and L2A-OE-MSCs were treated with or without TNF-α plus IFN-γ (10 ng/mL each) for 24 h. Protein, mRNA, and supernatant were collected. iNOS expression was measured by immunoblotting **a**, **d** and quantitative real-time PCR **b**, **e**. The concentration of NO in the culture supernatant was detected by Griess reagent **c**, **f**. **g** MSCs were pretreated with mitomycin C (50 ng/mL) for 4 h and then cocultured with splenocytes labeled with CFSE in the presence of anti-CD3/CD28 antibodies for 3 days at a ratio of 1:20. L-NMMA (1 mM) was added to block iNOS activity. CD8^+^ and CD4^+^ T cells were stained for proliferation analysis by flow cytometry at the end of coculture, and the percentage of nonproliferating T cells is shown. The results are representative of three to six independent experiments and are presented as the mean ± s.e.m. Significant differences were analyzed by one-way ANOVA **g** or two-way ANOVA **a**–**f**, and are expressed as *P < 0.05, ***P < 0.001, and n.s., no significance.

MSCs exert direct inhibitory effects on T cells through the production of immunosuppressive molecules, such as NO, indoleamine 2,3-dioxygenase, prostaglandin E2, TGFβ, haem oxygenase 1, and leukaemia inhibitory factor. To further investigate the mechanism by which CMA inhibits MSC immunosuppression on T cells, we treated SCR-MSCs and L2A-KD-MSCs with IFN-γ plus TNF-α, and then examined the molecules that were affected by CMA. The results showed that among the immunosuppressive molecules, only iNOS, which is involved in the production of NO, was significantly increased in L2A-KD-MSCs (Fig. 4a, b). Accordingly, the concentration of NO in the L2A-KD-MSC supernatant significantly increased (Fig. 4c). In contrast, LAMP-2A overexpression inhibited the expression of iNOS and the production of NO (Fig. 4d, e, f). Furthermore, a functional study showed that NG-monomethyl-L-arginine (L-NMMA), a specific inhibitor of iNOS activity, reversed the enhanced suppressive effects of L2A-KD-MSCs on T cell proliferation (Fig. 4g). In conclusion, these data indicate that inhibition of CMA enhances the immunosuppressive capacity of MSCs mainly through upregulating iNOS expression.

### CMA inhibits inflammation-induced STAT1 and NF-κB activation

**Fig. 5:**
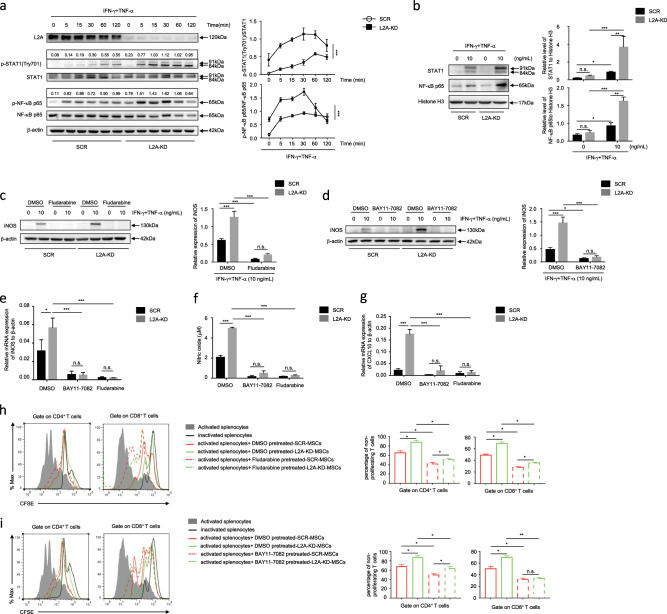
Inhibition of the STAT1 and NF-κB signaling pathways is responsible for the CMA-mediated inhibition of MSC immunosuppressive function. **a** SCR-MSCs and L2A-KD-MSCs were treated with or without TNF-α plus IFN-γ (10 ng/mL each) for the indicated time. Cells were harvested, and NF-κB p65, STAT1, and phosphorylation of NF-κB p65 and STAT1 at Tyr701 were analyzed by immunoblotting. The relative densitometry of p-NF-κB p65 and p-STAT1 (Tyr701) was quantified using ImageJ software and was compared to β-actin. **b** SCR-MSCs and L2A-KD-MSCs were treated with or without TNF-α plus IFN-γ (10 ng/mL each) for 6 h. Nuclear proteins were harvested. NF-κB p65 and STAT1 were analyzed by immunoblotting. **c**–**g** After pretreatment with DMSO, fludarabine (2 μM), or BAY11-7082 (10 μM) for 6 h, SCR-MSCs and L2A-KD-MSCs were treated with or without TNF-α plus IFN-γ (10 ng/mL each) for 24 h. Protein, mRNA, and supernatant were collected. The expression of iNOS was measured at the protein, and mRNA levels using immunoblotting and quantitative real-time PCR, respectively **c**–**e**. The concentration of NO in the culture supernatant was detected by Griess reagent **f**. CXCL10 mRNA expression was detected by quantitative real-time PCR **g**. **h**, **i** SCR-MSCs and L2A-KD-MSCs were pretreated with DMSO, fludarabine (2 μM), or BAY11-7082 (10 μM) for 6 h and then treated with mitomycin C (50 ng/mL) for 4 h prior to coculture with CFSE-labeled splenocytes activated by anti-CD3/CD28 antibodies for 3 days at a ratio of 1:20. CD8^+^ and CD4^+^ T cells were stained for proliferation analysis by flow cytometry at the end of coculture, and the percentage of nonproliferating T cells is shown. The results are representative of three to six independent experiments and are presented as the mean ± s.e.m. Significant differences were analyzed by one-way ANOVA **h**, **i** or two-way ANOVA **a**–**g**, and are expressed as *P < 0.05, ***P < 0.001, and n.s., no significance.

The NF-κB and STAT1 pathways are critical signals for IFN-γ− and TNF-α-induced iNOS and CXCL10 expression.^18^ We first performed immunoblotting to evaluate inflammatory cytokine-induced NF-κB activation upon CMA intervention in MSCs. As shown in Fig. 5a, b, LAMP-2A knockdown dramatically promoted the phosphorylation of NF-κB p65 and the nuclear translocation of p65 in MSCs after IFN-γ plus TNF-α stimulation. Moreover, IFN-γ plus TNF-α-induced phosphorylation of STAT1 at Tyr701 and enhanced nuclear STAT1 translocation in L2A-KD-MSCs (Fig. 5a, b). Functionally, pretreatment with the NF-κB inhibitor BAY11-7082^19^ or the STAT1 inhibitor fludarabine^20^ reversed the increased iNOS expression (Fig. 5c, d, e) and NO production (Fig. 5f), as well as CXCL10 expression (Fig. 5g) in L2A-KD-MSCs. As a result, the enhanced suppressive effects on T cell proliferation by L2A-KD-MSCs were abolished upon treatment with BAY11-7082 or fludarabine (Fig. 5h, i).

We applied pyrrolidinedithiocarbamate ammonium (PDTC),^20^ an inhibitor of IκB phosphorylation, which blocs NF-κB translocation to the nucleus and reduces the expression of downstream cytokines, and nifuroxazide,^21^ an inhibitor that suppresses the activation of cellular STAT1 transcriptional activity, to verify the role of NF-κB and STAT1 in CMA-mediated inhibition of MSC immunosuppressive capacity. Consistent with the effect of BAY11-7082 and fludarabine, PDTC and nifuroxazide reduced the expression of iNOS (Supplementary Fig. 2a, b) and CXCL10 (Supplementary Fig. 2c), and weakened the immunosuppression capacity of L2A-KD-MSCs (Supplementary Fig. 2d, e).

These data suggest that the inhibition of CMA promotes NF-κB and STAT1 activation, which in turn enhances iNOS and CXCL10 expression, and the immunosuppressive capacity of MSCs.

### Inflammatory factor-stimulated AKT activation mediates CMA inhibition

**Fig. 6:**
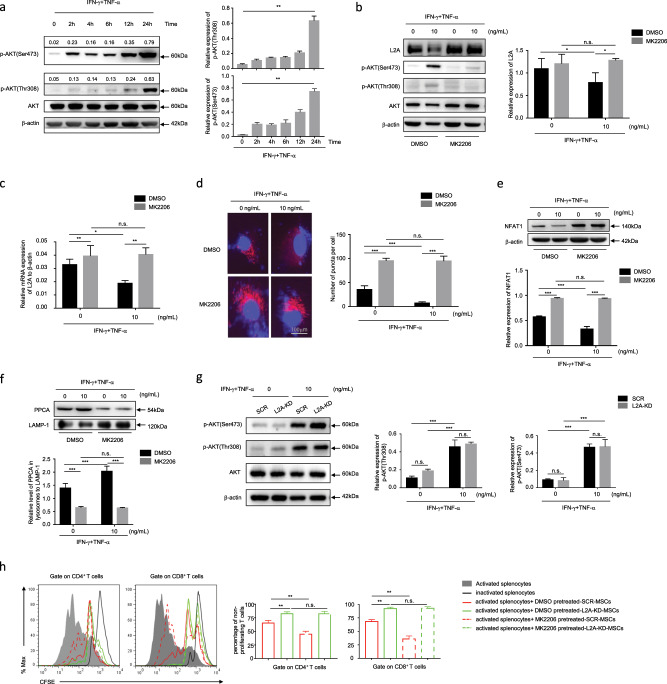
Inflammatory factor-stimulated AKT activation mediates CMA inhibition. **a** MSCs were treated with TNF-α plus IFN-γ (10 ng/mL each) and mRNA, and protein were collected at 0 h, 2 h, 4 h, 6 h, 12 h, and 24 h after treatment. AKT and AKT phosphorylation at Ser 473 and Thr 308 were analyzed by immunoblotting. **b**, **c** After pretreatment of MSCs with DMSO or MK2206 (10 μM) for 6 h, MSCs were treated with or without TNF-α plus IFN-γ (10 ng/mL each) for 24 h. Protein and mRNA were collected to detect the expression of L2A by immunoblotting **b** and quantitative real-time PCR **c**, respectively. **d** MSCs stably expressing KFERQ-PA-mCherry-C1 were photoactivated by a 405-nm light and then pretreated with DMSO or MK2206 for 6 h prior to treatment with TNF-α plus IFN-γ (10 ng/mL each) for 24 h. The images were collected by a Zeiss fluorescence microscope. The number of red fluorescent puncta per cell was quantified. **e** The level of NFAT1 was detected by immunoblotting using the proteins in **b**, and the quantitative analysis result is shown. **f** Lysosomes were isolated from MSCs that were pretreated with DMSO or MK2206 for 6 h and then treated with or without TNF-α plus IFN-γ (10 ng/mL each) for 24 h. Protein was extracted from the lysosomes, and the level of PPCA was measured by immunoblotting. The relative level of PPCA to LAMP-1 was calculated. **g** SCR-MSCs and L2A-KD-MSCs were treated with or without TNF-α plus IFN-γ (10 ng/mL each) for 24 h. Protein was collected, and AKT and phosphorylation of AKT at Ser 473 and Thr308 were analyzed by immunoblotting. **h** SCR-MSCs and L2A-KD-MSCs were pretreated with MK2206 or DMSO for 6 h and then treated with mitomycin C (50 ng/mL) for 4 h prior to coculture with CFSE-labeled splenocytes activated by anti-CD3/CD28 antibodies for 3 days at a ratio of 1:20. CD8^+^ and CD4^+^ T cells were stained for proliferation analysis by flow cytometry at the end of coculture, and the percentage of nonproliferating T cells is shown. Scale bar: 100 μm. The results are representative of three to six independent experiments and are presented as the mean ± s.e.m. Significant differences were analyzed by one-way ANOVA **a, h** or two-way ANOVA **b**–**g**, and are expressed as *P < 0.05, **P < 0.01, ***P < 0.001, and n.s., no significance.

AKT signaling has been widely investigated in the proliferation, differentiation, and survival of MSCs,^22–24^ but few reports have shown whether AKT signaling participates in the regulation of MSC immunosuppression. We found that the phosphorylation level of AKT at Ser473 and Thr308 increased following IFN-γ plus TNF-α stimulation with time (Fig. 6a). As AKT activation on lysosomes was reported to suppress the activation of CMA,^25^ we wondered whether proinflammatory factor-induced AKT activation contributed to CMA inhibition in MSCs. We then blocked the activation of AKT using the phosphorylation inhibitor MK2206^26^ and LY294002, which inactivates AKT signaling through inhibiting phosphoinositide 3-kinase.^27^ As expected, the decreased LAMP-2A expression caused by IFN-γ plus TNF-α recovered and even increased when AKT activation was inhibited (Fig. 6b, c, Supplementary Fig. 3a, b). The CMA reporter experiment showed the same results, as the number of red fluorescent puncta increased after MK2206 was added (Fig. 6d). Mechanistically, we found that when AKT activation was blocked, the level of nuclear factor of activated T cells 1 (NFAT1), which is reported to upregulate the expression of LAMP-2A,^4^ was markedly elevated even in the presence of IFN-γ plus TNF-α (Fig. 6e, Supplementary Fig. 3c). Moreover, the degradation rate of LAMP-2A was also reduced with MK2206 inactivation of AKT, as demonstrated by the decreased PPCA in lysosomes isolated from MK2206-pretreated MSCs (Fig. 6f). Consistent with the aforementioned results, these data indicate that proinflammatory factors inhibit CMA activity via AKT signaling, and AKT signaling activation decreases NFAT1, leading to the reduced expression and accelerated the degradation of LAMP-2A.

Then, we wondered whether CMA affects the activation of AKT similar to the effects on NF-κB and STAT1. The results showed that CMA did not affect the phosphorylation of AKT, as evidenced by the fact that the phosphorylation of AKT at both Ser473 and Thr308 exhibited no significant difference between the LAMP-2A knockdown MSCs and the scramble MSCs (Fig. 6g).

Furthermore, functional experiments revealed that pretreatment with MK2206 or LY294002 significantly inhibited the immunosuppressive effect of MSCs on T cell proliferation, when compared with that of the dimethyl sulfoxide (DMSO)-pretreated MSCs but had no influence on L2A-KD-MSCs (Fig. 6h, Supplementary Fig. 3d).

Collectively, these data indicate that inflammation-induced AKT activation is responsible for the inhibition of CMA in MSCs.

### CMA inhibition improves the therapeutic effects of MSCs in inflammatory liver injury

**Fig. 7:**
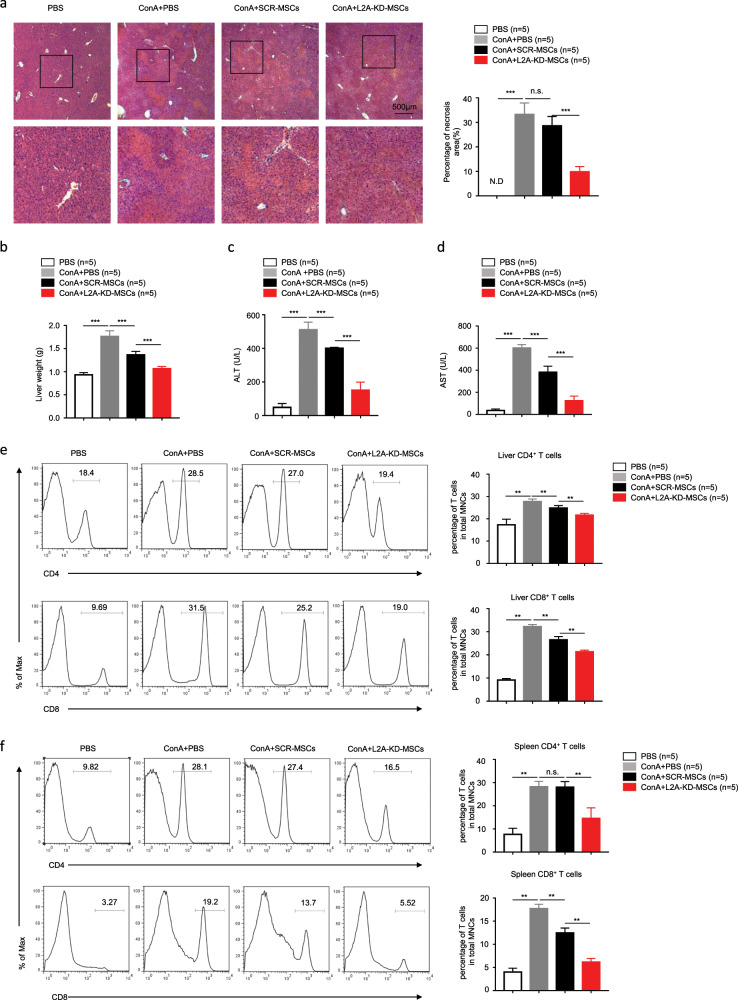
Knockdown of LAMP-2A improves the therapeutic effects of MSCs on ConA-induced inflammatory liver injury. Mice were intravenously injected with ConA (20 mg/kg) or PBS as a negative control, and then SCR-MSCs and L2A-KD-MSCs were transfused immediately. Eight hours later, the livers, spleens, and serum were sampled. **a** Hematoxylin and eosin staining of liver sections. The percentages of necrotic areas in the livers were calculated (*n* = 5 mice per group). **b** The weight of the livers was measured (*n* = 5 mice per group). **c**, **d** Serum levels of ALT and AST were measured by the enzyme-linked immunosorbent assay (*n* = 5 mice per group). **e**, **f** The percentages of CD8^+^ and CD4^+^ T cells in the liver and spleen were determined by flow cytometry, and the statistical results are shown (*n* = 5 mice per group). Scale bar: 500 μm. The results are presented as the mean ± s.e.m. Significant differences were analyzed by one-way ANOVA **a**–**f** and are expressed as *P < 0.05, **P < 0.01, ***P < 0.001, and n.s., no significance.

MSCs are one of the most potent stem cells used in the clinical treatment for inflammation-related diseases because of their unique immunosuppressive capacity and multilineage differentiation ability. To date, MSCs have been applied to treat a variety of mouse disease models, such as experimental autoimmune encephalomyelitis^28^ and graft-versus-host disease,^29^ and favorable therapeutic effects have been obtained. Concanavalin A (ConA)-induced liver injury is a well-established murine model of T-cell-mediated hepatitis. Intravenous injection of ConA induces acute liver injury and systemic immune activation in mice, which resembles the pathology of immune-mediated hepatitis in humans.^30^ Several studies have applied MSCs to treat this inflammatory lesion, but the effect was not satisfactory.^31^ Consistent with the published data, the curative effect of SCR-MSCs was poor. As expected, L2A-KD-MSCs exhibited an improved therapeutic effect on liver injury, as shown by decreased centrilobular necrosis (Fig. 7a) and reduced liver weights (Fig. 7b). Consistently, reduced serum alanine aminotransferase (ALT) and aspartate transaminase (AST) levels were detected in mice administered L2A-KD-MSCs (Fig. 7c, d). Next, we detected the number and percentage of CD8^+^ and CD4^+^ T cells both in the liver and spleen obtained from the different treatment groups. As shown in Fig. 7e, f, the percentages of CD8^+^ and CD4^+^ T cells in the liver and spleen from mice administered L2A-KD-MSCs were much lower than those of the control groups.

We further established another inflammatory liver injury model induced by *Propionibacterium acnes* (*P. acnes*) to verify the enhanced immunosuppression of L2A-KD-MSCs.^32^ In this model, numerous mononuclear cells (MNCs) accumulate in the liver and secrete proinflammatory cytokines, which promote the activation and proliferation of CD4^+^ T cells and liver inflammation.^26^ Consistent with the ConA model, we tested the lesions of the liver after therapeutic treatment with PBS, SCR-MSCs, or L2A-KD-MSCs, as well as the liver weight, levels of ALT and AST, and the infiltration of CD4 + and CD8 + T cells in the spleen and liver. We found that the treatment with L2A-KD-MSCs markedly reduced the granuloma formation and infiltration of lymphocytes (Supplementary Fig. 4a, b). The spleens of the L2A-KD-MSC-treated group were also smaller than those of the SCR-MSC-treated group, indicating low-grade inflammation (Supplementary Fig. 4a). These results were consistent with a dramatic decrease in ALT and AST levels in the serum of L2A-KD-MSC-treated mice (Supplementary Fig. 4c, d). The liver weights were also significantly reduced in the L2A-KD-MSC-treated group (Supplementary Fig. 4e). Moreover, the total number of infiltrating monocytes in the liver and spleen, as well as the percentage of CD8^+^ and CD4^+^ T cells, were calculated, and the results were similar to those of the ConA-induced model (Supplementary Fig. 4f, g, h).

Taken together, these data indicate that applying CMA-deficient MSCs to an inflammatory liver injury model significantly alleviates inflammation, thus improving the therapeutic effect of MSCs in liver injury.

## Discussion

CMA, which is one type of autophagy, maintains cellular homeostasis in starvation and controls protein quality under stress. The selective degradation of specific proteins endows CMA with tight regulation of unique cellular processes. Here, we found that CMA plays an essential role in the immunosuppression of MSCs induced by inflammatory factors. Our data showed that in response to the inflammatory factors TNF-α and IFN-γ, CMA was inhibited in MSCs, as manifested by decreased LAMP-2A expression at the mRNA and protein levels. LAMP-2A depletion, which effectively blocks CMA, enhanced the immunosuppression of MSCs on the proliferation of CD8^+^ and CD4^+^ T cells via increasing the expression of CXCL10 and iNOS, thus improving the therapeutic effects of MSCs in inflammatory liver injury.

It has been well characterized that in inflammatory environments, MSCs exert immunosuppressive effects on immune cells (e.g., T cells, B cells, dendritic cells, and macrophages) via molecular mechanisms, thereby being useful in treating various degenerative and inflammatory disorders.^5^ However, little is known about the mechanisms by which inflammatory factors regulate the immunosuppression of MSCs. Our previous work indicated that inflammatory factors activate MSC macroautophagy, which inhibits the immunosuppressive function of MSCs, thus impairing their therapeutic effects in inflammatory disorders. It has been reported that the impairment of macroautophagy leads to the activation of CMA under both basal and stressful conditions,^10^ and compensatory CMA provides a protective effect against the dysfunction of cells due to compromised macroautophagy.^11^ Likewise, CMA blockage also induces macroautophagy upregulation, but macroautophagy only partially compensates for CMA functions.^33^ Macroautophagy and CMA communicate with each other extensively, and one can serve as a backup mechanism when the other is depressed. We have proven that in an inflammatory environment, macroautophagy is activated;^9^ thus, it is reasonable to hypothesize that CMA is deactivated. As expected, under the stimulation of inflammatory factors, CMA was inhibited in MSCs, especially with prolonged stimulation, because of the reduced synthesis and accelerated degradation of LAMP-2A. More interestingly, the suppressed CMA exerted a beneficial function in the immunosuppressive capacity of MSCs on T cell proliferation, as manifested by the enhance immunosuppression of L2A-KD-MSCs and inhibited immunosuppression of L2A-OE-MSCs. This is consistent with macroautophagy-mediated inhibition of MSC immunosuppression, as we previously demonstrated. Our present work provides further evidence that macroautophagy and CMA are interconnected to influence cellular function.

Various immune pathways contribute to MSC-mediated immunosuppression. Among them, the chemokine–iNOS axis was the first discovered and is the main mechanism involved in the interaction between MSCs and inflammatory factors, which subsequently leads to the inhibition of T cell proliferation.^12^ The majority of chemokines produced by inflammatory factor-activated MSCs are CXC-chemokine receptor 3 and CC-chemokine receptor 5 ligands, including CXCL9, CXCL10, CXCL11, and CCL5, which are well-known chemoattractants for T cells. In addition, the upregulation of iNOS in MSCs results in the increased production of NO in the surroundings of MSCs, leading to cell cycle arrest and inhibited proliferation in T cells. Because we discovered that CMA negatively regulated the immunosuppression of MSCs on T cell proliferation, we hypothesized that the chemokine–iNOS axis may participate in this process. We found that more splenocytes or A1.1 cells were recruited to LAMP-2A-modified MSCs than to scrambled MSCs. Transwell migration assays indicated that factors secreted by cytokine-activated MSCs recruited the splenocytes. Among these chemokines, CXCL9, CXCL10, CXCL11, and CCL5 recruit T cells and are secreted by MSCs, but only CXCL10 expression was significantly increased even under basal conditions and reached a very high level with inflammatory factor stimulation in CMA-compromised MSCs. In addition, LAMP-2A knockdown in MSCs significantly promoted iNOS expression and NO production, while overexpression of LAMP-2A exerted the opposite effects. Moreover, NO depletion via L-NMMA reversed the enhanced immunosuppression of L2A-KD MSCs. These results suggest that CMA negatively regulates the expression of both CXCL10 and iNOS. In inflammatory environments, CMA activation is inhibited, and so this inhibitory effect on CXCL10 and iNOS by CMA is relieved, promoting the immunosuppressive function of MSCs. Because MSCs pervasively exist in bone marrow, adipose tissue, muscle, and many other tissues, this inhibitory effect of CMA on CXCL10 expression may play an important role in protecting these tissues from inflammatory damage.

Our results further revealed the molecular mechanisms underlying CMA-mediated inhibition of CXCL10 and iNOS, and the resultant inhibition of MSC immunosuppression. STAT1 and NF-κB are two main signaling pathways that participate in inflammation-induced CXCL10^34^and iNOS^18^expression. NF-κB inhibition also results in decreased iNOS in IFN-γ-treated MSCs,^35^ and when STAT1 activation is inhibited, the increased iNOS expression in MSCs induced by inflammation vanishes.^36^ In our study, we found that the level and duration of STAT1 and NF-κB activation were significantly increased in CMA-compromised MSCs. In addition, after inhibition of these two signals using specific inhibitors, the increased expression of CXCL10, iNOS, and NO production resulting from altering CMA was abolished. These results suggest that CMA inhibition-mediated immunosuppression of MSCs is dependent on the deactivation of STAT1 and NF-κB signaling.

CMA is involved in regulating cell functions through degrading specific proteins, and it is very important and necessary to discover the substrate proteins undergoing degradation in CMA-mediated inhibition of STAT1 and NF-κB. It has been reported that IκBα, which is an inhibitor of NF-κB, is a substrate of CMA. However, our results showed that there was no significant difference in the protein level of IκBα between L2A-KD-MSCs and scramble-MSCs. Moreover, this is contradictory because IκBα degradation is beneficial for the activation of NF-κB, but CMA inhibits NF-κB activation. Therefore, IκBα is not a specific substrate protein in CMA-mediated STAT1 and NF-κB inhibition in inflammation-primed MSCs. We next screened key molecules in these two signaling pathways and found that both IκB kinase α (IKKα) and IKKβ have the KFERQ-like motif (data not shown), suggesting that these IKKs may also be CMA substrates. IKK promotes NF-κB activation by phosphorylating IκBα, which results in translocation of p65 into the nucleus. If CMA degrades IKKs, NF-κB signaling would be inhibited, since unphosphorylated IκBα binds p65 and inhibits its translocation, which is consistent with the results we obtained. We then measured the level of IKKα and IKKβ in SCR-MSCs and L2A-KD-MSCs treated with/without IFN-γ plus TNF-α. The results indicated that IKK-α may be the target protein of CMA in CMA-mediated inhibition of NF-κB signaling (Supplementary Fig. 2f). However, further work is required to elucidate and validate the exact substrate proteins in CMA-mediated inhibition of MSC immunosuppression.

Our results showed that upon inflammatory factor stimulation, AKT was activated in MSCs. Other studies indicated that AKT signaling is involved in the regulation of MSC survival, proliferation, differentiation, apoptosis, and migration.^22–24,37,38^ Wu T et al. noted that activation of PTEN/AKT signaling further activates NF-κB to promote TGFβ1 expression, thereby enhancing the immunosuppressive capacity of MSCs.^39^ We hypothesized that CMA also regulates the activity of the AKT signaling pathway. However, there was no difference in AKT activation between L2A-KD-MSCs and scramble-MSCs, and this result indicates that CMA does not regulate AKT activity in MSCs. According to a recent study, phosphorylation of AKT on the lysosomal membrane inhibits the activity of CMA by promoting dissociation of the protein channels composed of LAMP-2A.^25^ Based on this study, we hypothesized that inflammatory factor-induced AKT activation is responsible for CMA inhibition. Stimulation with MK2206/LY294002, which inhibits AKT phosphorylation, significantly increased the expression of LAMP-2A, and the degradation rate of LAMP-2A decreased, which enhanced CMA activity even in inflammatory environments. The increased level of NFAT1 likely explains the elevated expression of LAMP-2A due to AKT inhibition. These results indicate that AKT activation caused by inflammation inhibits CMA activity, promoting MSC immunosuppression. However, further study is required to explore the underlying mechanism through which AKT inhibits CMA activity.

MSCs are unique adult stem cells that have both stem cell properties and immunomodulatory function, are easy to amplify in vitro, and have little antigenicity. All of these characteristics make MSCs an effective tool for the treatment of autoimmune and inflammatory diseases. Currently, a large number of studies and clinical trials have used MSCs to treat inflammatory diseases, such as liver disease (hepatitis, liver failure, etc.), graft-versus-host disease, experimental autoimmune encephalomyelitis, and inflammatory bowel disease.^28,29,32,40^ A ConA-induced inflammatory liver injury mouse model is a well-established model of liver damage induced by acute immune responses, in which T lymphocytes have been identified as the major effector cells.^30^Several studies have reported that human tonsil and murine adipose tissue-derived MSCs can ameliorate the development of ConA-induced inflammatory liver injury.^41,42^ Han et al. first found that murine bone morrow MSCs had no effect on this acute inflammatory disease model, while interleukin-17-pretreated MSCs exhibited enhanced therapeutic effects.^31^ In the present study, scramble-MSCs had poor therapeutic effects on ConA-induced inflammatory liver injury, while L2A-KD-MSCs notably alleviated this inflammatory disease. In addition, mice induced with *P. acnes* also had acute liver injury mediated by inflammatory infiltration and immune responses. In this model, T cells play an important role in the progression of liver damage.^26^ Our previous work described a therapeutic method to treat this inflammatory disease using MSCs and obtained a reasonable effect.^32^ In the present study, we found that, CMA-deficient MSCs exhibited a better curative effect on liver injury with decreased inflammation compared to that of SCR-MSCs. These data tell us that CMA-deficient MSCs have enhanced immunosuppression, resulting in better therapeutic effects.

In conclusion, we found that inflammation-induced CMA inhibition, which further maintains the immunosuppressive function of MSCs. The present study not only enriched the basic knowledge of the immunoregulatory plasticity of MSCs but also provided a new intervention target to improve the clinical therapeutic efficacy of MSCs.

## Materials and methods

### Reagents and mice

Recombinant murine IFN-γ and TNF-α were purchased from PeproTech (Rocky Hill, USA). Antibodies against β-actin, iNOS, NF-κB p65, phospho-NF-κB p65, IκBα, histone H3, STAT1, phospho-STAT1 (Tyr701), AKT, phospho-AKT (Ser473), phospho-AKT (Thr308), LAMP1, PPCA, and LAMP-2s (ABL93) were purchased from Cell Signaling Technology (Danvers, USA). Antibodies against LAMP-2A, HSPA8, NFAT1, and IKKα+β were purchased from Abcam (San Francisco, USA). L-NMMA, Fludarabine, MK2206, PDTC, Nifuroxazide, and LY294002 were purchased from Selleckchem (Houston, USA). BAY11-7082 was purchased from MedChemExpress (Shanghai, China). ConA, Griess reagents, and the lysosome isolation kit were from Sigma (St Louis, USA). C57BL/6 mice were purchased from the Shanghai Laboratory Animal Center of the Chinese Academy of Sciences and maintained under specific pathogen-free conditions in the vivarium of the Shanghai Institute of Nutrition and Health of the Chinese Academy of Sciences. All animal experiments were performed according to the guidance of the Institutional Animal Care and Use Committee of the Shanghai Institutes for Biological Sciences of the Chinese Academy of Sciences, and complied with the Guide for the Care and Use of Laboratory Animals published by the U.S. National Institutes of Health.

### Cells

Murine bone marrow MSCs were isolated as previously described.^9,32^ Briefly, murine MSCs were generated from the bone cavity of femurs and tibias of 3- to 4-week-old C57BL/6 mice. The cells were cultured in low glucose DMEM containing 10% fetal bovine serum, 2 mM L-glutamine, and 1% penicillin–streptomycin (All from Life Technologies GmbH). A single-cell suspension of bone marrow cells was seeded in 100 mm culture dishes, non-adherent cells were removed after 24 h, and the medium was replenished every 2 days. Cells were used at the 9–16th passages. The stemness of murine MSCs was determined by their expression of specific cell surface markers and by their capability to differentiate into adipocytes, osteoblasts, and chondrocytes. The MSCs used in each experiment were paired and at the same passage. HEK 293 T cells were purchased from ATCC (Manassas, USA).

### Quantitative real-time polymerase chain reaction

Total RNA was extracted with TRIzol (Life Technologies GmbH) and reverse-transcribed into cDNA with the reverse transcription kit from TaKaRa (Tokyo, Japan). The mRNA levels were measured by real-time polymerase chain reaction (PCR) with SYBR Green reagent from Roche (Natley, USA) and normalized to the mRNA level of β-actin. Primer sequences were as follows: mouse β-actin, sense 5′- CCACGAGCGGTTCCGATG-3′ and antisense 5′-GCCACAGGATTCCATACCCA-3′; LAMP-2A, sense 5′- AGGTGCTTTCTGTGTCTAGAGCGT-3′ and antisense 5′- AGAATAAGTACTCCTCCCAGAGCTGC-3′; mouse iNOS, sense 5′-TGGAGCGAGTTGTGGATTGT-3′ and antisense 5′-GGGTCGTAATGTCCAGGAAGTA-3′; mouse CXCL9, sense 5′-TGCTACACTGAAGAACGGAGATC-3′ and antisense 5′-CTTCCTTGAACGACGACGACT-3′; mouse CXCL10, sense 5′-GTAAGCTATGTGGAGGTGCG-3′ and antisense 5′-GGAAGATGGTGGTTAAGTTCG-3′; mouse CXCL11, sense 5′TGTAATTTACCCGAGTAACGGC-3′ and antisense 5′- CACCTTTGTCGTTTATGAGCCTT-3′; and mouse CCL5, sense 5′-GCTGCTTTGCCTACCTCTCC-3′ and antisense 5′-TCGAGTGACAAACACGACTGC-3′.

### Immunoblot analysis

Cells were harvested and lysed in radioimmunoprecipitation assay buffer (Beyotime, Haimen, China) containing phenylmethylsulfonyl fluoride (Beyotime) for 30 min on ice. Lysates were clarified by centrifugation at 15,000 × *g* for 30 min. The protein concentration of the supernatant fraction was determined by a Bradford assay (Thermo Fisher Scientific, New Hampshire, USA). Protein samples were diluted in 4 × sodium dodecyl sulfate loading buffer (TaKaRa), heated to 95 °C for 5 min and fractionated in a 10% Sodium dodecyl sulfate–polyacrylamide gel. Proteins were electroblotted onto a polyvinylidene fluoride and incubated for 1 h in 5% bovine serum albumin in Phosphate-buffered saline (PBS) or nonfat dry milk dissolved in PBS containing 0.1% Tween-20 (PBST) at room temperature. The blotting membranes were incubated with primary antibodies overnight at 4 °C, extensively washed in PBST, incubated with horseradish peroxidase-conjugated secondary antibody (Cell Signaling Technology) for 1 h at room temperature, and washed again with PBST. The blotting membranes were developed with chemiluminescent reagents (Millipore, Billerica, USA) according to the instructions provided by the manufacturer.

### Lentiviral vector construction

Oligonucleotides with the following nucleotide sequences were used for the cloning of shRNA-encoding sequences into the lentiviral vector PLKO.1 puro, which was a gift from Bob Weinberg (Addgene, Cambridge, USA): mouse LAMP-2A: sh1-LAMP-2A, 5′-CCGGGACTGCAGTGCAGATGAAGCTCGAGCTTCATCTGCACTGCAGTCTTTTTG-3′; sh2-LAMP-2A, 5′-CCGGCTGCAATCTGATTGATTACTCGAGTAATCAATCAGATTGCAGTTTTTG-3′; sh3-LAMP-2A, 5′-CCGGAAACACTGCTTGACCACCCTCGAGGGTGGTCAAGCAGTGTTTTTTTTG-3′; and scrambled control (scramble), 5′-CCGGCCTAAGGTTAAGTCGCCCTCGCTCGAGCGAGGGCGACTTAACCT TAGGTTTTTG-3′; (synthetized by Sangon Biotech). High titer lentiviral stocks were produced, and murine MSCs at the 9th passage were infected with scrambled control lentivirus (scramble-MSCs) or lentivirus expressing shRNA that inhibited LAMP-2A (sh1-LAMP-2A-MSCs, sh2-LAMP-2A-MSCs, and sh3-LAMP-2A-MSCs) according to the manufacturer’s protocol (http://www.addgene.org/tools/protocols/plko/). Cells that were resistant to puromycin (4 μg/mL) were selected at the 11th passage and were used at the 14–16th passages.

### Overexpression

Full-length mouse LAMP-2A cDNA was synthesized by GenScript (Nanjing, China). These cDNAs were subcloned into pLVX-IRES-zsGreen vectors. Murine MSCs were transfected with pLVX-IRES-zsGreen or pLVX-IRES-zsGreen containing LAMP-2A by using Lipofectamine 2000 (Life Technologies GmbH).

### KFERQ-PA-mCherry1 reporter assay

Oligonucleotides with the following nucleotide sequence was used for the cloning of KFERQ sequences into the lentiviral vector PA-mCherry-C1, which was a gift from Michael Davidson (Addgene, USA): KFERQ: 5′-AATTCTGCAGTCGACGGTACCGCGGGC CC-3′. MSCs were stably infected with PA-KFERQ-mCherry-C1 lentivirus. For CMA activity analysis, cells were photoactivated by a 405/20-nm LED array, followed by treatment, and were then fixed and costained with 4′,6-diamidino-2-phenylindole. The images were acquired using a Zeiss fluorescence microscope and subjected to deconvolution using MATLAB. The activation of CMA activity was calculated as the number of cells that presented bright puncta under a 20 × objective and was quantified by using ImageJ software (NIH).

### In vitro T cell proliferation assay

Murine MSCs were pretreated with mitomycin C (50 ng/mL) to inactivate MSCs by inhibiting their proliferation while reserving their immunosuppressive capacity and were then seeded into 48-well plates. Freshly isolated splenocytes (4 × 10^5^ cells/well) from C57BL/6 mice were labeled with 5 μM carboxyfluorescein diacetate succinimidyl ester (CFSE, Thermo Fisher Scientific) and cocultured with murine MSCs for 3 days in the presence of mouse anti-CD3/CD28 antibodies (eBiosciences, San Diego, USA) The cells were then collected for flow cytometric analysis on a fluorescence-activated cell sorting (FACS) Calibur flow cytometer (BD Biosciences, Franklin Lakes, USA).

### Griess assay

Fifty microliters of culture supernatant of different treated murine MSCs and standards were added to a flat bottomed 96-well plate, and then 50 μL of Griess reagent (Sigma) was added. The absorbance was measured at 540 nm after incubation for 15 min in the dark at room temperature, and the NO concentrations were calculated.

### Isolation of MSC lysosomes

An enriched lysosomal fraction from MSCs with different treatments was isolated by differential centrifugation followed by density gradient centrifugation using a lysosome isolation kit (Sigma-Aldrich). The detailed procedure was carried out according to the product instructions.

### ConA-induced inflammatory liver injury in mice

Male C57BL/6 mice (8–10 weeks old) were intravenously injected with ConA in PBS at 20 mg/kg to induce inflammatory liver injury. Murine MSCs (1 × 10^6^) were intravenously administered to mice immediately after ConA injection. The mice were killed, and serum, spleen, and liver tissues were sampled 8 h after ConA injection. Four percent paraformaldehyde-fixed liver histological sections were stained with hematoxylin and eosin. Liver MNCs were purified by a Percoll gradient, spleen MNCs were purified by a Ficoll gradient, and all MNCs were stained with anti-CD4 and anti-CD8a (eBiosciences) for 30 min at 4 °C in staining buffer and then analyzed by flow cytometry on a FACSCalibur flow cytometer (BD Biosciences).

### *P. acnes*-induced liver injury mouse model

C57BL/6 mice were primed intravenously with 1 mg of heat-killed *P. acnes*. For the indicated experiments, a total of 1 × 10^6^ SCR-MSCs, L2A-KD-MSCs, or PBS was injected intravenously on days 3, 5, and 7 after injection of *P. acnes*. On day 7, the mice were sacrificed, and serum, spleen, and liver tissues were sampled. Photos of the spleen and liver from the different groups were taken, and the liver weights were recorded. Four percent paraformaldehyde-fixed liver histological sections were stained with hematoxylin and eosin. Liver MNCs were purified by a Percoll gradient, spleen MNCs were purified by a Ficoll gradient, and all MNCs were calculated and stained with anti-CD4 and anti-CD8a (eBiosciences) for 30 min at 4 °C in staining buffer before analysis by flow cytometry on a FACSCalibur flow cytometer (BD Biosciences).

### Statistical analysis

All data are shown as the mean ± s.e.m. and were obtained from at least three independent experiments. Significant differences were analyzed by Mann–Whitney *U* test, one-way analysis of variance (ANOVA), and two-way ANOVA with GraphPad Prism (version 7.0, GraphPad Software). Significance was expressed as *P < 0.05, **P < 0.01, and ***P < 0.001.

## Supplementary information

supplementary figure

Supplementary Movie 1

Supplementary Movie 1a

Supplementary Movie 1b
