# Supplementary material for: Inflammation-induced inhibition of chaperone-mediated autophagy maintains the immunosuppressive function of murine mesenchymal stromal cells
Source: Cell Mol Immunol. 2020 Jan 3;18(6):1476–88. doi: 10.1038/s41423-019-0345-7 (PMC8167126; doi:10.1038/s41423-019-0345-7)

Supplementary Figure 1. Inhibition of CMA promotes the immunosuppression of MSCs but has no effect on MSCs proliferation.

a MSCs were transfected with lentivirus consisted of four different plasmids expressing scramble-RNA (SCR) and shRNA1 (L2A-KD1), shRNA2 (L2A-KD2), shRNA3 (L2A-KD3) specific to LAMP-2A. mRNA of different treated MSCs was collected, and LAMP-2A expression was detected by quantitative real-time PCR. **b** L2A-KD-MSCs and SCR-MSCs were pretreated with mitomycin C (50 ng/mL) for 4h, and then cocultured with CFSE-labeled A1.1 cells for 3 days in the presence of anti-CD3/CD28 antibodies at the indicated ratios. A1.1 cells were collected for proliferation analysis by flow cytometry at the end of coculture and the percentage of inactivated A1.1 cells were shown. **c** The proliferation capacity of SCR-MSCs and L2A-KD-MSCs was detected by CCK8 at the indicated time points after cell passaging. **d** The clone formation capacity of SCR-MSCs and L2A-KD-MSCs were presented by crystal violet staining. Scale bar: 1mm. Results are representative of six to eight independent experiments and presented as mean ± SEM. Significant differences were analyzed by Mann–Whitney U test (**b**), Two-way ANOVA (**c**), and expressed as: ***P< 0.001.

Supplementary Figure 2. Inhibitors of STAT1 and NF-κB attenuate the enhanced immunosuppression of MSCs due to CMA deactivation.

a-c After pretreatment with DMSO or PDTC (10 μM) or Nifuroxazide (10 μM) for 6h, SCR-MSCs and L2A-KD-MSCs were treated with or without TNF-α plus IFN-γ (10 ng/mL respectively) for 24h. Protein and mRNA were collected. The expression of iNOS was measured at protein and mRNA levels using immunoblotting analysis and quantitative real-time PCR (**a, b**) And CXCL10 expression was detected by quantitative real-time PCR at mRNA level (**c**). **d, e** SCR-MSCs and L2A-KD-MSCs were pretreated with DMSO or PDTC (10 μM) or Nifuroxazide (10 μM) for 6h and then treated with mitomycin C (50 ng/mL) for 4h prior to coculture with CFSE-labeled splenocytes activated by anti-CD3/CD28 antibodies for 3 days at a ratio of 1:20. $CD8^+$ and $CD4^+$ T cells were stained for proliferation analysis by flow cytometry at the end of coculture and the percentage of non-proliferating T cells were shown. **f** SCR-MSCs and L2A-KD-MSCs were treated with or without TNF-α plus IFN-γ (10 ng/mL respectively) for 24h. Cells were harvested and IKKα and IKKβ were

analyzed by immunoblotting analysis. The relative densitometry of IKKα and IKKβ was quantified respectively using ImageJ software compared to β-actin. Results are representative of three independent experiments and presented as mean ± SEM. Significant differences were analyzed by One-way ANOVA (**d, e**), Two-way ANOVA (**b, c, f**), and expressed as: ***P*< 0.01, ****P*< 0.001, n.s. no significance.

Supplementary Figure 3. Ly294002 increases the expression of LAMP-2A resulting in depressed immunosuppression of MSCs.

a-c After pretreatment of MSCs with DMSO or Ly294002 (10 μM) for 6h, MSCs were treated with or without TNF-α plus IFN-γ (10 ng/mL respectively) for 24h. Protein and mRNA were collected to detect the expression of L2A by immunoblotting analysis (**a**) and quantitative real-time PCR (**b**). And the level of NFAT1 was measured by immunoblotting (**c**). **d** SCR-MSCs and L2A-KD-MSCs were pretreated with LY294002 (10 μM) or DMSO for 6h and then treated with mitomycin C (50 ng/mL) for 4h prior to coculture with CFSE-labeled splenocytes activated by anti-CD3/CD28 antibodies for 3 days at a ratio of 1:20. $CD8^+$ and $CD4^+$ T cells were stained for proliferation analysis by flow cytometry at the end of coculture and the percentage of non-proliferating T cells were shown. Results are representative of three independent experiments and presented as mean ± SEM. Significant differences were analyzed by One-way ANOVA (**d**), Two-way ANOVA (**a, b**), and expressed as: *P< 0.05, **P< 0.01, ***P< 0.001, n.s. no significance.

Supplementary Figure 4. CMA deficiency improves the therapeutic effect of MSCs on *P.acnes*-induced inflammatory liver injury.

Mice were intravenously injected with heat-killed *P.acnes* (1mg), PBS as negative control, and then SCR-MSCs, L2A-KD-MSCs were transfused at days 3, 5, 7 after the *P.acnes* injected. At day 7, mice were sacrificed, and livers, spleens, serum were sampled. **a** Livers and spleens from different groups were photographed. The representative images were showed. Scale bar: 1cm **b** Hematoxylin and eosin staining of liver sections. Scale bar: 200μm. **c, d** Serum levels of ALT and AST were measured by ELISA. **e** The weight of livers was weighed. **f** Total number of MNCs from liver and spleen was calculated. **g, h** The percentages of $CD8^+$ and $CD4^+$ T cells in liver and spleen tissues were determined by flow cytometry and the statistical results were shown. n=3 (**c-h**) mice in PBS treated group, n=8

(**c-h**) mice in *P.acnes*+ PBS treated group, n=8 (**c-h**) in *P.acnes* + SCR-MSCs treated group, n=6 (**c, d**)/ n=8 (**e-h**) in *P.acnes* + L2A-KD-MSCs treated group. Results are presented as mean ± SEM. Significant differences were analyzed by One-way ANOVA (**c-h**) and expressed as: *P< 0.05, **P< 0.01, ***P< 0.001, n.s. no significance.

Supplementary Figure 1.

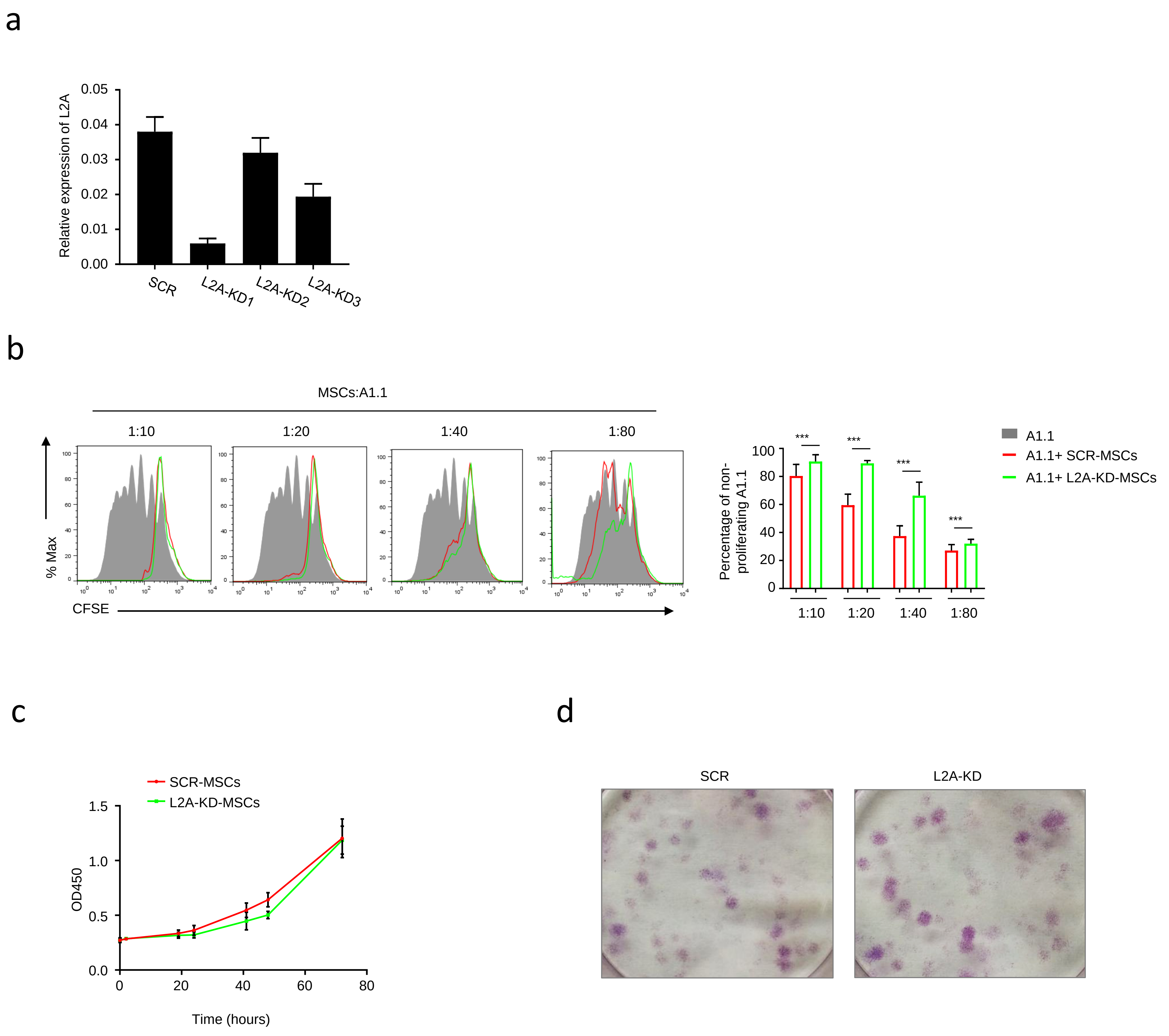

Supplementary Figure 2.

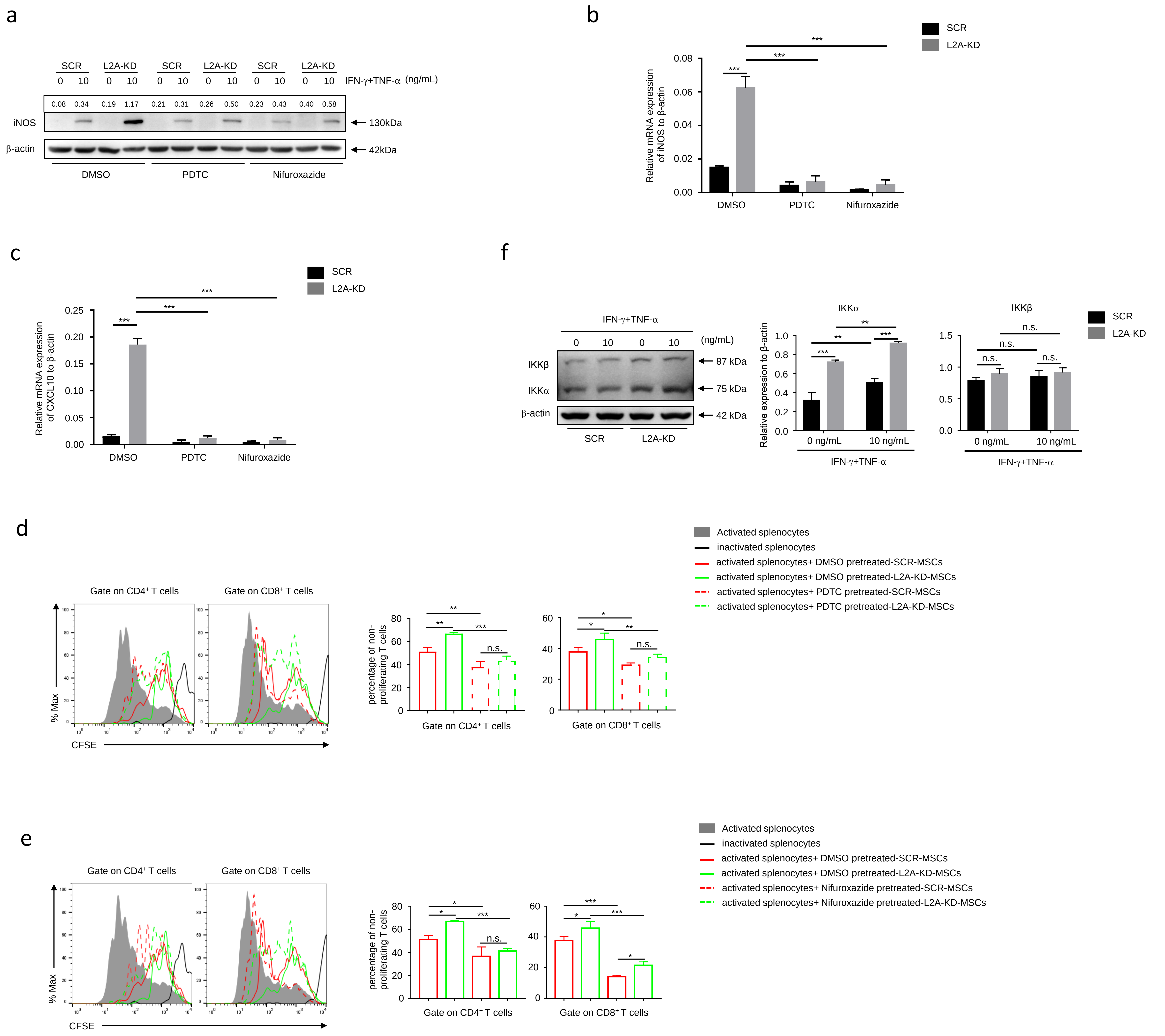

Supplementary Figure 3.

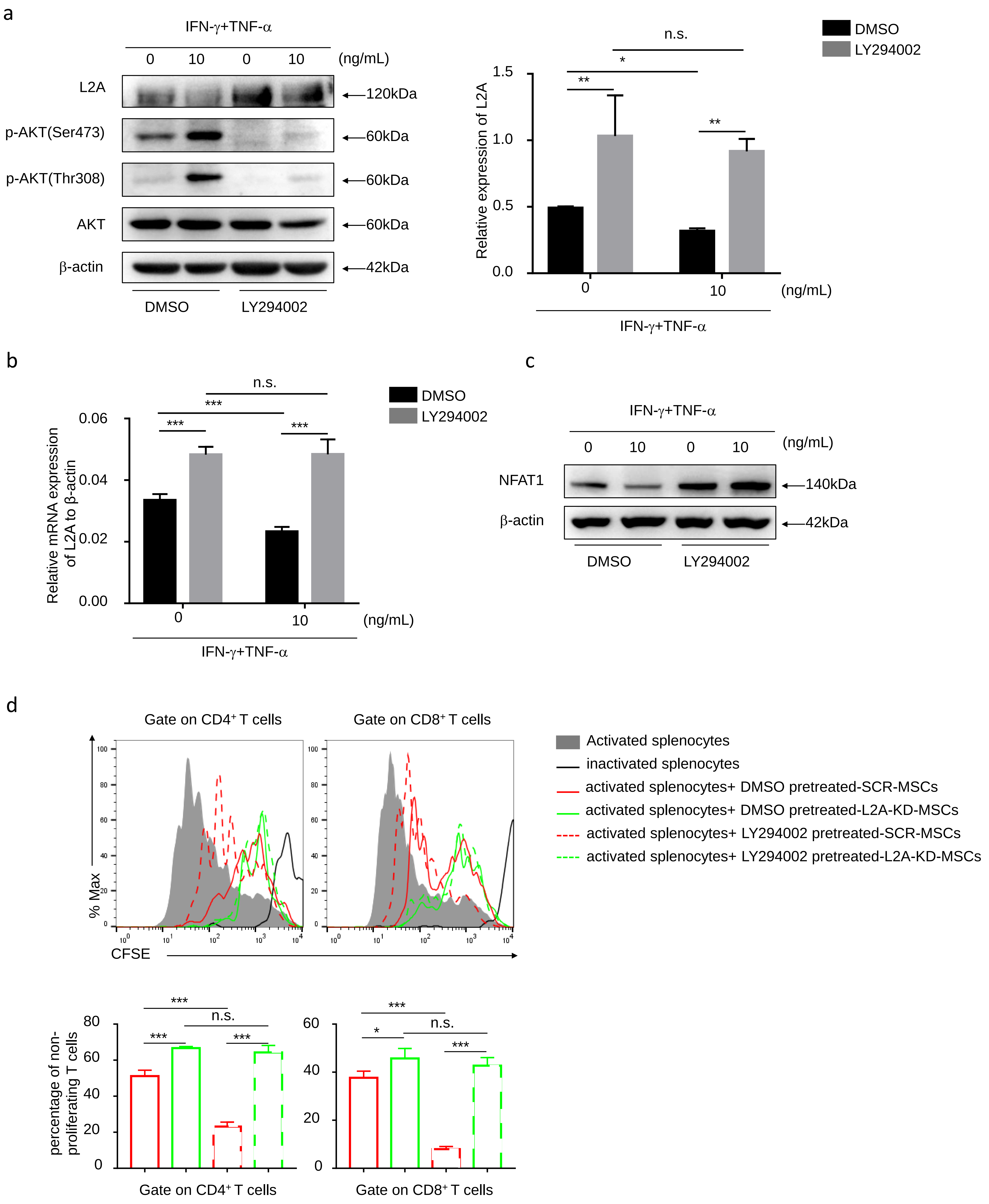

Supplementary Figure 4.

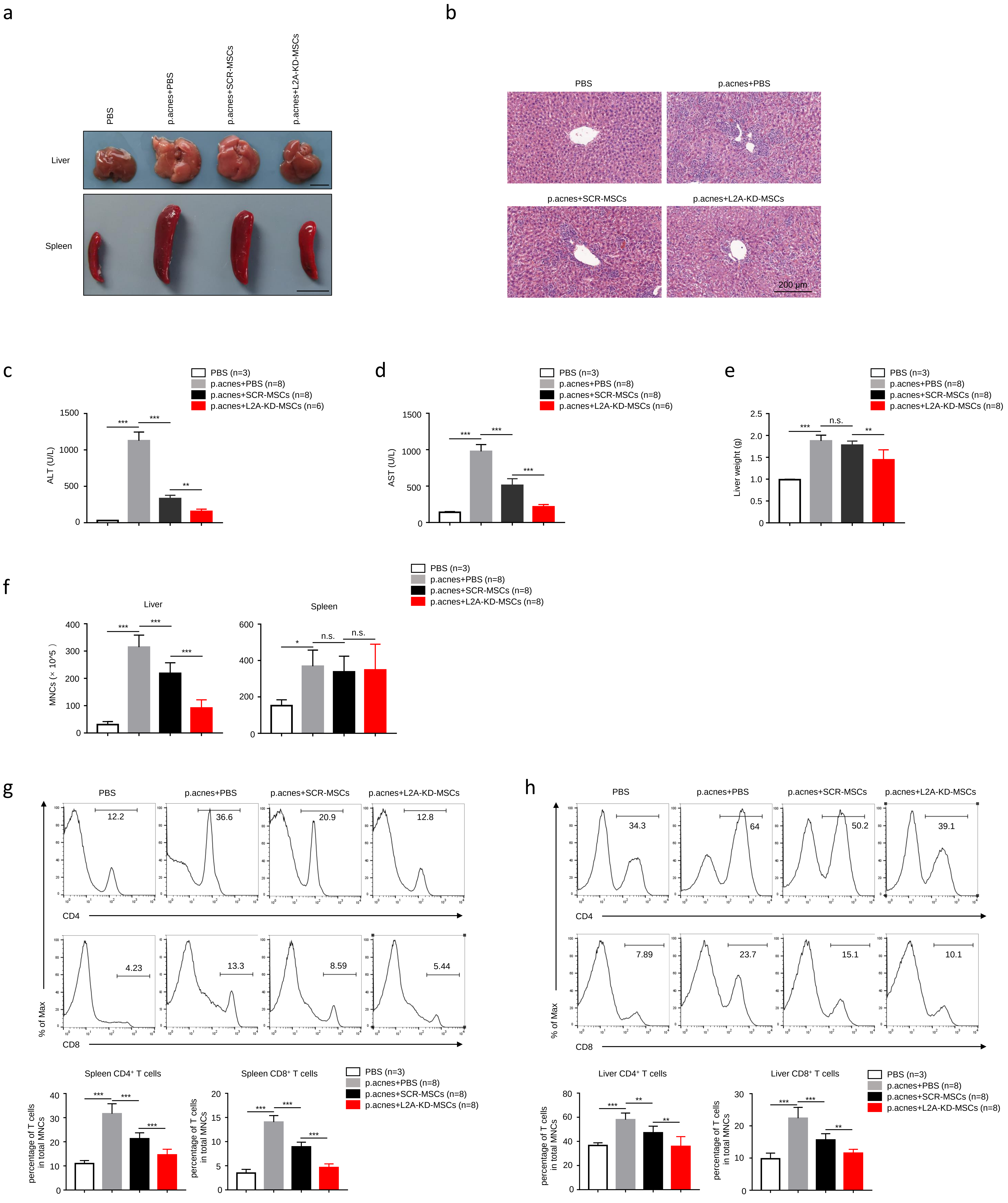

Supplement: Supplementary file 1 — supplementary figure [file 41423_2019_345_MOESM1_ESM.pdf]
