# Supplementary material for: Inflammation-induced inhibition of chaperone-mediated autophagy maintains the immunosuppressive function of murine mesenchymal stromal cells
Source: Cell Mol Immunol. 2020 Jan 3;18(6):1476–88. doi: 10.1038/s41423-019-0345-7 (PMC8167126; doi:10.1038/s41423-019-0345-7)
Supplement: Supplementary file 2 — Supplementary Movie 1 [file 41423_2019_345_MOESM2_ESM.pdf]

Supplementary Movie 1

Mitomycin pretreated SCR-MSCs (**Supplementary Movie 1a**) and L2A-KD MSCs (**Supplementary Movie 1b**) were cocultured with splenocytes in the presence of anti-CD3/CD28 antibodies. Took images every 30min within 12h of coculture initiation by Operetta High-Content Imaging System.
